# Tuning of Reciprocal Carbon‐Electrode Properties for an Optimized Hydrogen Evolution.

**DOI:** 10.1002/cssc.202100654

**Published:** 2021-05-07

**Authors:** Yuxiao Ding, Liyun Zhang, Qingqing Gu, Ioannis Spanos, Norbert Pfänder, Kuang Hsu Wu, Robert Schlögl, Saskia Heumann

**Affiliations:** ^1^ Max Planck Institute for Chemical Energy Conversion Stiftstrasse 34–36 45470 Mülheim an der Ruhr Germany; ^2^ Qufu Normal University 57 Jingxuan West Road Qufu 273165 P. R. China; ^3^ School of Chemical Engineering University of New South Wales Kensington Sydney NSW 2052 Australia; ^4^ Fritz-Haber-Institut der Max-Planck Gesellschaft Faradayweg 4–6 14195 Berlin Germany

**Keywords:** biomass, carbon electrode, carbon oxidation, chemical energy conversion, water splitting

## Abstract

Closing the material cycle for harmful and rare resources is a key criterion for sustainable and green energy systems. The concept of using scalable biomass‐derived carbon electrodes to produce hydrogen from water was proposed here, satisfying the need for sustainability in the field of chemical energy conversion. The carbon electrodes exhibited not only water oxidation activity but also a strong self‐oxidation when being used as anode for water splitting. The carbon oxidation, which is more energy‐favorable, was intentionally allowed to occur for an improvement of the total current, thus enhancing the hydrogen production on the cathode side. By introducing different earth‐abundant metals, the electrode could be well adjusted to achieve an optimized water/carbon oxidation ratio and an appreciable reactivity for practical applications. This promising methodology may become a very large driver for carbon chemistry when waste organic materials or biomass can be converted using its intrinsic energy content of carbon. Such a process could open a safe path for sub‐zero CO_2_ emission control. The concept of how and which parameter of a carbon‐based electrode can be optimized was presented and discussed in this paper.

## Graphical abstract

**Self‐oxidation**: The concept of using carbon‐based electrodes to produce hydrogen from water is proposed. The electrodes exhibit not only good water oxidation activity but also a more energy‐favorable self‐oxidation in water splitting, fulfilling an energy conversion of carbon (theoretical 100 %) and electricity into hydrogen.

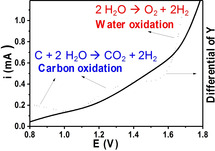

## Introduction

The energy challenge can be seen as the major challenge for today's society and future generations.^[1]^ The direct consumption of electricity from renewable sources like solar or wind is a sustainable way for easing the pressure of fossil‐energy systems, but the daily or seasonal dependent production does not directly follow the needs of the population. The conversion and storage of the related electrical energy into hydrogen, a chemical energy carrier with high energy density, becomes the most promising way.^[2]^ Water is a sustainable source of green hydrogen. Therefore, water splitting to produce hydrogen is a critical process.^[3]^ In this process, the oxidation of water at the anode is still challenging due to the high overpotential, and the associated instability of the anode materials under those harsh conditions.^[4]^ Noble metal oxides such as IrO_2_ and RuO_2_ require lower energy barriers for the water oxidation process so that hydrogen can be generated at the cathode with appreciable rate at relatively low applied voltages.^[5]^ However, the noble metal‐based catalysts are costly and their supply is not sustainable, which restricts their scalable implementation.

Although some of first‐row transition metals‐based materials exhibit appreciable water oxidation reactivity,[^2d^, ^3^, ^6^] their intrinsically insufficient electrical conductivity requires efficient charge transport by conductive support materials like metals or carbons such as graphene and carbon nanotubes.^[7]^ Furthermore, due to their environmental acceptability, high surface area, and excellent electron transfer ability, carbon materials (especially heteroatom‐doped ones) are recently being used as metal‐free catalysts for the anodic reaction.^[8]^ However, whether as supports or as catalysts, the influence from the carbon structure is inevitable. The complexity of carbon structures and the varying influence on different properties complicate the understanding of these compound materials. This also leads to a lot of controversial conclusions, despite strong statements in the literature. In addition, although the electrocatalytic activity of carbon materials could enable them to be used potentially as metal‐free catalysts, the stability of carbon materials at reaction conditions is rarely investigated.^[9]^ The effect of carbon corrosion is often seen in literature by a slight increase in potential before the oxygen evolution reaction (OER) region, but not much attention is drawn to this.^[10]^ It is worth noting that the potential for carbon oxidation (C+H_2_O→CO_2_+4H^+^+4e^−^) is 0.207 V vs. standard hydrogen electrode (SHE).^[11]^ That means weakly bonded carbon atoms within the carbon structure might also be effortlessly oxidized during the water splitting at the potential lower than the water oxidation process and provide a more efficient way to produce hydrogen. This strategy of a sacrificial electrode is discussed in detail in our previous work.^[12]^

In a popular direction, plant biomass has been used to produce low‐cost and scalable carbonaceous materials,^[13]^ filling an important role in the carbon circulation. Herein, we developed a series of biomass‐derived carbons that were operated in the water splitting process, by using glucose as representative for biomass for a knowledge‐based synthesis. The potential of chemical modification by introduction of metal species as well as thermal treatment to tune the electrocatalytic performance are investigated in detail. With this work we want to demonstrate how complex the properties are connected with each other and that for an optimization a compromise of reciprocal properties has to be found.

## Results and Discussion

**Figure 1:**
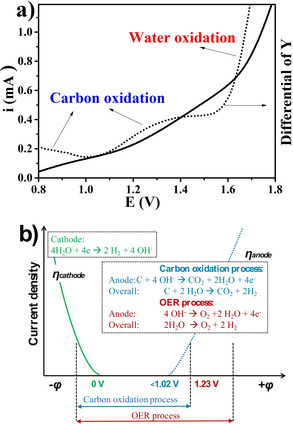
(a) Potential–current curve of the carbon sample (C600) as electrode and the relative differential curve. (b) Electrochemical polarization curves of water splitting when use carbon as sacrificial electrode. The inset are related reaction equations of both OER and carbon oxidation.

The carbon samples were synthesized by hydrothermal treatment of glucose solutions in Teflon‐lined autoclaves and subsequent pyrolysis (details in the Supporting Information and Figure S1).^[14]^ A film of as‐synthesized sample was dropcoated onto a glassy carbon (GC) electrode for different electrochemical measurements in Ar‐saturated 0.1 m KOH (total loading: 50 μg cm^−2^, details in the Supporting Information). A reversible hydrogen electrode (RHE) was used as reference electrode for all performed electrochemical measurements. Figure 1a shows the oxidation curve from a carbon sample (C600, the number represents the annealing temperature of 600 °C in Ar; this applies for the whole paper), from which the oxidation behavior of carbon is exhibited. The peaks of the differentiated curve (the dashed line) reveal the carbon oxidation processes (the first oxidation process at around 0.8 V might be the formation of oxygen functional groups on the carbon surface and the second one from around 1.0 V might be the formation of CO_2_) and the water oxidation process (onset potential is at around 1.55 V). Figure 1b shows the polarization curves of both anode and cathode sides. The carbon oxidation and water oxidation process share the same cathode reaction to produce hydrogen. Apparently, with a standard potential lower than 1.0 V, the oxidation and consumption of the carbon at the anode require much lower energy to produce hydrogen than the water oxidation, which needs a standard potential of 1.23 V. Therefore, the scalable carbon material can be used as a sacrificial electrode for water splitting with less energy consumption, in which the energy of carbon oxidation is theoretically 100 % converted into hydrogen. The carbon structure and its stability hereby determine where the onset potential at the anode is. The plan for CO_2_ production at the anode is to recycle it naturally back into biomass where it came from according to the concept of a sacrificial biodegradable electrode.^[12]^ Additionally, carbonate can be obtained as the product in the alkaline, which is a very beneficial industrial process (using KOH to produce K_2_CO_3_).

**Figure 2:**
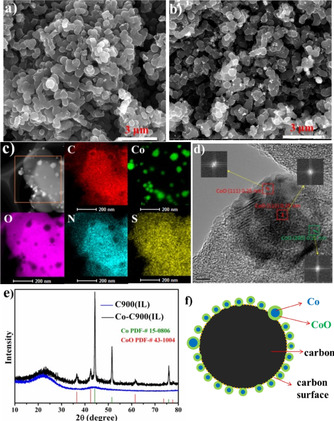
SEM images of (a) C900 and (b) Co−C900(IL). (c) EDX mapping images of different elements and (d) HRTEM image of Co−C900(IL). (e) XRD curves of C900(IL) and Co−C900(IL). (f) Schematic image of the sample.

The metal‐free carbon requires only low potential to enable the reaction, but due to the kinetic limitation, the reaction rate is relatively slow. Doping the carbon structures with different earth‐abundant metals or heteroatoms influences the performance of the electrodes. Natural biomass sources already contain relevant dopants. To mimic their natural contributions from biomass and to reach desirable reaction rates at the anode a simple doping method of just mixing and grinding carbon and metal precursor solutions is proposed here to get a uniform distribution of the dopants. The functionalized carbon can then be obtained by subsequent annealing. Different solvents like water or ionic liquids (IL) were used for the preparation of metal solutions. IL were applied to achieve a high dispersity, as it can also be achieved with natural biomass sources, where metals are often present in a complexed form. In addition, the IL contain heteroatoms, which makes them good candidates for simple and local doping. In this study as representative for many other possibilities, cobalt acetate dissolved in an IL (1‐butyl‐3‐methylimidazolium hydrogen sulfate) as metal precursor to produce a cobalt doped sample, was used. Figure 2a,b displays the scanning electron microscopy (SEM) images of the pure carbon sample C900 and the cobalt doped carbon sample Co−C900(IL), respectively. The morphology of the carbon backbone has no obvious change with the introductions of the dopants. The bright spots on the carbon surface are from the cobalt species. Energy‐dispersive X‐ray spectroscopy (EDX) mapping images in Figure 2c show that all elements originating from the IL (N, S, O) are homogeneously distributed on the carbon surface. This confirms that the IL is a good candidate for a homogeneous introduction of heteroatoms onto the carbon surface. Based on the non‐color‐filled parts with Co particle shapes in different elemental images, it can be assumed that Co has a metallic core structure. The metallic species are probably formed due to the reducing effects of carbon at high temperatures. On the high‐resolution transmission electron microscopy (HRTEM) images of the sample (Figure 2d), only CoO lattices can be observed, which means that the particles have most likely a CoO@Co core–shell structure. X‐ray diffraction (XRD, Figure 2e) analyses also confirm that Co and CoO crystalline species coexist beside the carbon support and no further crystalline phases can be detected. Figure 2f shows a schematic image of the obtained sample. The dopants from IL and cobalt precursor are introduced in the synthesis procedure after the formation of the spherical carbon precursor, therefore they are distributed mainly on the carbon surface. The grinding process before annealing introduces a homogeneous distribution of the different elements on the carbon surface (Figure S2). The annealing process unavoidably leads to the formation of some aggregated cobalt species, but the homogeneous distribution of non‐metallic dopants maintains due to the physical adsorption of the IL on the carbon surface.^[15]^

**Figure 3:**
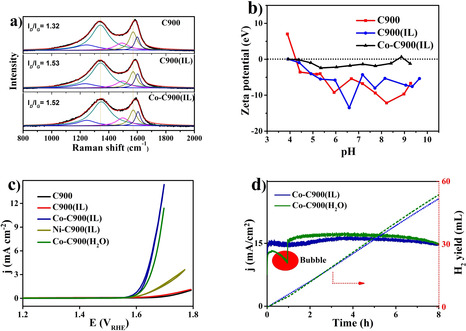
(a) Raman curves of C900, C900(IL), and Co−C900(IL) and applied 5 peak fitting according to amorphous materials. (b) Zeta potential of HTC900, HTC900(IL), and CoHTC900(IL). (c) CV of different samples with a scan rate of 5 mV min^−1^. (d) CA test of cobalt contained samples at 1.8 V and the calculated hydrogen yield from the measured current.

Raman spectroscopy is one of the most used characterization techniques to analyze carbon structures.^[16]^ The entities from molecular vibrations overlap in the Raman spectra with the lattice vibrations of graphitic structures (G, D, and D′) making the precise determination impossible for these biomass derived carbons. Yet, the comparison of the different samples in Figure 3a still demonstrates the introduction of both IL and the Co has no obvious influence on the carbon structure. This can also be confirmed by the XPS results (Figure S3), where the C 1 s spectra of different samples with and without Co‐species overlap perfectly. The surface functional groups on the C900 sample can be protonated and deprotonated depending on the pH value resulting in positively or negatively charged Zeta potentials. The C900(IL) and C900 samples show consistently negative potentials in the tested pH with a slight tendency towards neutral values at low pH (Figure 3b). The Co−C900(IL) has a zeta potential close to zero throughout the whole investigated pH range, which means that cobalt species depolarize the carbon surface by interacting with the surface functional groups.

Figure 3c shows the cyclic voltammetry (CV) curves from different samples with *iR* correction after conditioning. Metal‐containing samples Co−C900(IL) (the cobalt is introduced in IL solution), Co−C900(H_2_O) (the cobalt is introduced in water solution), and Ni−C900(IL) show much better activity than the two metal‐free samples (C900(IL) and C900). Especially the two Co‐containing samples show acceptable activity for practical application. As a conclusion, the activity results mainly from the Co species on the carbon surface. The key performance indicator (KPI) of the electrochemical data of the two samples are given in Tables S1 and S2. To determine the stability of Co−C900(IL), a chronoamperometric (CA) measurement was performed by maintaining a constant potential at 1.8 V for 8 h (Figure 3d). The stability of the sample remained constant for 8 h. Upon the accumulated quantity of electric charge, the theoretical amount of H_2_ under atmospheric pressure was calculated. The measured amount of produced H_2_ after 8 h H_2_ is 52 mL. The Co−C900(H_2_O) shows a similar stability at the same potential of 1.8 V. The comparison of the two samples indicates that the heteroatoms from the IL have no obvious contribution to the activity and stability of the metal species. However, a reproducible glitch of the Co−C900(H_2_O) appears due to the accumulation of gas bubbles on the electrode surface during the reaction. For the heteroatom‐enriched Co−C900(IL) surface, the gas can be directly released without bubble formation. This demonstrates the important effect of heteroatoms for improving the aqueous affinity of the carbon surface. This observation is supported by the previous work, which demonstrated stronger interactions with water and hydrothermal carbon surfaces when they contain nitrogen.^[17]^ Furthermore, the heteroatoms lead to a higher capacity of the sample (Figure S4). Reasons for the deactivation may be due to the degradation of either the Co species or the carbonaceous support. Both ex‐situ and in‐operando inductively coupled plasma optical emission spectroscopy (ICP‐OES) measurements (Supporting Information) detect no Co solubility during the stability tests of different samples, which means the degradation of the activities is most likely caused by the consumption of the carbon support.

**Figure 4:**
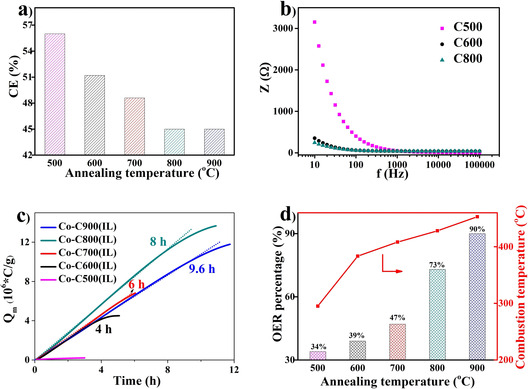
(a) Carbonization efficiency (CE) of precursor mixtures at different temperatures. (b) Electrochemical impedance spectroscopy at the open circuit potential of different samples. (c) Quantity of electric charge (*Q*) accumulation curves with different samples at 1.8 V. Tangent lines on each curve represent the stable current periods. (d) OER percentage obtained by in‐situ detecting oxygen production by an oxygen sensor and combustion temperatures under air (details in the Supporting Information) of different samples.

Considering that the carbon structures have big influence on the carbon oxidation process, varied carbon structures are obtained by changing the annealing temperatures (related samples are named as C500, C600, C700, C800, and C900 depending the annealing temperatures). Generally, 600 °C is considered as the temperature when carbonization starts to happen.^[15b]^ Figure 4a shows the carbonization efficiency (calculation details in the Supporting Information) of the precursors at different temperatures. The carbonaceous materials still experience a condensation process when the annealing temperature is lower than 800 °C. The condensation leads to obvious weight loss forming connected five‐ or six‐membered rings with localized π structure.[^15b^, ^18^] When the annealing temperature is higher than 800 °C, the condensation process is almost finished. Further increase of the temperature proceeds in a structure organization and a start of graphitization. During this step, the weight loss is not evident. From XRD patterns of different metal‐free samples, no graphitic (002) reflection peak can be observed, only a broad C (101) reflection at 43.5° increases with increasing annealing temperature (Figure S5). This indicates that carbonaceous structures assemble and become more organized when the annealing temperature exceeds 600 °C, but no graphitization occurred at temperature lower than 900 °C. The carbonaceous sp^2^ carbon network is indispensable for a good conductivity by endowing a delocalized π structure. Accordingly, the sample annealed at 500 °C shows a much higher resistance at low frequency (3125 Ω at 10 Hz, Figure 4b). After forming a carbonaceous network (annealed after 600 °C), the resistances of different samples become equivalent (around 300 Ω at 10 Hz). The high resistance of the sample annealed at 500 °C originates from the high amount of organic sp^3^ components, which limit the electron transfer.

When a cobalt precursor is involved during the annealing process, aggregation of the Co species takes place. The process is slower at lower temperatures, introducing lower crystal peak intensities in the XRD pattern (Figure S6). The fitting of the Raman spectra of the cobalt containing samples show a decrease of the amorphous part with increase of the annealing temperature (Figure S7). The species are still Co and CoO, but the size of the particles becomes much smaller, which also introduces a more uniform distribution of the particles compared to the Co−C900(IL) (Figure S8). The better distribution of the Co species does not result in better activity; on the contrary, with decreasing annealing temperature, both activity and stability decrease. Figure 4c shows the charge accumulation curves of different samples at 1.8 V. Due to the high concentration of the sp^3^ carbon structure, the sample annealed at 500 °C has almost no charge contribution. With the annealing temperature of 600, 700, 800, and 900 °C, the samples show constant charge accumulations over time 4, 6, 8, and 9.6 h, respectively. Interestingly, the annealed sample at 800 °C has a steeper slope than the sample annealed at 900 °C, which means it has an enhanced activity. This effect can be explained by a higher carbon oxidation contribution. For the sample annealed at 800 °C, around 73 % of the current are consumed by water oxidation and 27 % for carbon oxidation (Figure 4d). Although most carbon atoms have conductive sp^2^ character (with delocalized π electrons), carbon atoms at defect and edge sites with lower bonding energies are more easily oxidized and contribute to the carbon oxidation part. Therefore, the samples with more defects and edges contribute to hydrogen production mainly via carbon oxidation. With the increase of the annealing temperature from 600 to 900 °C, the carbon oxidation current decrease from 61 to only 10 %. The carbon oxidation process can be described as a mild combustion. When the “combustion” starts, more active sites will be exposed from the relative stable structures. This process introduces more oxidizing sites over time and explains why there is an initial activation step in the CA test (Figure S9), while in Figure 3d, the 900 °C annealed sample has almost no initial activation. Furthermore, the combustion process is easier in samples with less developed sp^2^ carbon structures, which also results in a faster decomposition of the samples (Figure S9). The same combustion procedure occurs during annealing in air. With an increase of the annealing temperature during synthesis from 500 to 900 °C under argon atmosphere, the combustion temperatures of the different samples increase from 295 to 453 °C (Figures 4d, S10). Additional to the annealing temperature, the annealing time also has an impact on the degree of the carbonization. A longer annealing time results in higher activities and higher stabilities (Figure S11).

The kinetic instability of electrodes is still a core challenge in the field of water splitting. In alkaline media, even commercial IrO_*x*_ electrodes show non‐satisfying stability.^[19]^ Therefore, if the degradation of the anodes cannot be eliminated, a sacrificial electrode could be considered as a solution. Biomass derived carbon is a perfect candidate for an industrial‐scalable source due to its renewability, low cost and the environmental acceptability. Considering that the surface area of the electrode is around 0.2 cm^2^, the total loading content of the catalyst on the glassy carbon electrode is 10 μg. For the 900 °C annealed sample, 10 μg catalyst produces 540 C of electric charge and 2.8 mmol H_2_ will be produced within 9.6 h. Under the same electrochemical conditions, the 800 °C annealed sample can produce 3.5 mmol H_2_. This demonstrates that the reactivity of the electrode can be optimized towards an improved water oxidation and carbon oxidation ratio and that the reciprocal properties need to be tuned wisely. The results are quite promising at this low scale. This work was not designed to determine the optimum but rather to show the way to it. When the loading is increased, a longer time of stable current can be obtained (Figure S12). The optimization between the carbon oxidation and water oxidation is dependent on the cost of the carbon and the requirements from industry including parameter like the electrode changing and the used electricity. This indicator will dictate the synthesis procedure of optimized annealing temperatures and time for the preparation. To make a more robust electrode, a pellet technique is currently ongoing in our group to meet the requirements of a large‐scale application. It is also worth to mention that the real stability of the anode materials is much better, because in the current case ink corrosion contribute partly to the degradation.

## Conclusion

A new concept of converting carbon and electricity energy into hydrogen with the water splitting process is proposed. Biomass‐derived carbon was used as sacrificial agent to produce a constant current at the anode, while at the cathode hydrogen was continuously formed. A chemical modification by introduction of metal species highly improved both oxygen evolution and carbon oxidation activity. By an increase of the annealing temperature and the annealing time of the carbon samples, their electrocatalytic performance increases. The reason is that the high temperature and the long time lead to a decrease of the amorphous entities, edges and defects, which are the sites that being oxidized first. By adjusting the balance between the reciprocal water oxidation and carbon oxidation, an optimized hydrogen production can be reached. The sources of carbon‐containing precursors are infinite from plants and food waste. This concept provides an industrially scalable and sustainable option to obtain hydrogen from water and also a substitution of the low‐efficiency carbon‐based fuel (like coal) combustion process.

## Experimental Section

### Synthesis of the materials

The synthesis of the precursor materials was performed in Teflon‐lined autoclaves heated in a heating block system to 220 °C for 6 h. For a typical experiment, 30 mL of a 20 wt% glucose solution were prepared in aqueous solution. The solution pH was 6. After the hydrothermal treatment, the autoclaves were taken out of the heating device and were allowed to cool down over night. The solid product was filtered and washed with water thoroughly. The solid product was dried in vacuum at 60 °C overnight. The final product was named as HTC and used as precursors for all the following annealing procedures. The obtained carbon precursor was moved to small quartz boats, which were placed in the center of a larger quartz tube running through the center of a furnace. Temperature‐programmed annealing processes were carried out according to the following procedures: annealing the hybrid from room temperature to 60 °C (Argon, 20 min), maintaining for 2 h; then heating to different temperatures with a heating rate of 10 K min^−1^, maintaining for 5 h. The oven was cooled down to room temperature without active cooling. The final products were obtained and named as C600 or C900 (the numbers represent the annealing temperatures). 400 mg HTC and 150 mg IL were mixed and ground to get a homogeneous mixture. The mixture was annealed accordingly to the temperature program for C900 to obtain C900(IL). 70 mg cobalt acetate (Sigma Aldrich, 99.995 %) and nickel acetate (Sigma Aldrich, 98 %) were dissolved in 150 mg IL before the solutions were mixed and grounded with 400 mg carbon precursor to get a homogeneous mixture. The mixtures then were annealed with same temperature programs as C900 to obtain Co−C900(IL) and Ni−C900(IL), respectively. 70 mg cobalt acetate was dissolved in 220 mg water and then mixed and ground with 400 mg carbon precursor. The mixture was annealed with the same temperature program as C900 to obtain CoHTC900(H_2_O).

### Characterization techniques

The TEM images and STEM‐EDX elemental mapping in Figure 2 were observed on a FEI Tecnai G2 F20 microscope operated at 200 kV. The mapping images in Figure S2 were detected by STEM Hitachi HD‐2700. XRD measurements were performed on a D/max 2400 diffractometer (JEOL Ltd, Japan) at a scanning rate of 4° min^−1^, with graphite monochromatized CuK_α_ radiation (*I*=0.1506 nm). Raman spectra were measured by a Thermo Scientific DXR Raman Microscope with a 50× magnification and a 532 nm laser. The electrochemical measurements were conducted in a three‐electrode system, which was controlled by using a potentiostat/galvanostat (BioLogic VSP, France). A platinized Pt wire as a counter electrode and a RHE (HydroFlex, Gaskatel GmbH) as a reference electrode were used.

## Conflict of interest

The authors declare no conflict of interest.

## Supporting information

As a service to our authors and readers, this journal provides supporting information supplied by the authors. Such materials are peer reviewed and may be re‐organized for online delivery, but are not copy‐edited or typeset. Technical support issues arising from supporting information (other than missing files) should be addressed to the authors.

Supplementary
