# Supplementary material for: Tuning of Reciprocal Carbon‐Electrode Properties for an Optimized Hydrogen Evolution
Source: ChemSusChem. 2021 May 7;14(12):2547–53. doi: 10.1002/cssc.202100654 (PMC8252440; doi:10.1002/cssc.202100654)
Supplement: Supplementary file 1 — Supplementary [file CSSC-14-2547-s001.pdf]

ChemSusChem

Supporting Information

Tuning of Reciprocal Carbon-Electrode Properties for an Optimized Hydrogen Evolution

Yuxiao Ding,* Liyun Zhang, Qingqing Gu, Ioannis Spanos, Norbert Pfänder, Kuang Hsu Wu, Robert Schlögl, and Saskia Heumann*© 2021 The Authors. ChemSusChem published by Wiley-VCH GmbH. This is an open access article under the terms of the Creative Commons Attribution License, which permits use, distribution and reproduction in any medium, provided the original work is properly cited.

The synthesis of samples at lower temperatures

The synthesis of samples at lower temperatures is consistent to the ones prepared at 900 °C. The only difference is that there was no dwell time because the samples were annealed at the same time and taken out when the temperature was reached. The related carbonization efficiencies of each sample are calculated with the weight left after annealing process with the following equation.

$$\text{Carbonization efficiency} = \frac{Weight\ after\ annealing}{Weight\ of\ the\ HTC\ before\ annealing} \times 100\%$$

Pretreatment of electrodes

Working electrode is 5 mm Ø glassy carbon (GC) disk electrode in PEEK sheath. Proper electrode pretreatment to get a mirror-like surface is done before every measurement. Before the first use, electrodes are sandpapered with decreasing roughness. Electrodes are polished with polishing paste (Al_2O_3 slurry; 1.0 µm and 0.05 µm) on a wet polishing cloth for 3-5 minutes, and rinsed thoroughly with water. No scratches are visible on the glassy carbon surface. Before drop-coating of catalyst, electrodes are ultrasonicated in absolute ethanol for 5 minutes, rinsed thoroughly with water, ultrasonicated in millipore water for 5 minutes, rinsed thoroughly with water and dried in an oven at 60 °C.

Ink preparation

Ink consists of 4 mL IPA, 960 µL H_2O and 40 µL Nafion solution (binder) and 5 mg sample. The catalyst is dispersed in the solvent by 15 min of ultrasonication. Proper dispersion yields a well dispersion without obvious particles. The catalyst precipitates from the dispersion over time. If any black precipitate can be seen, ultrasonication is repeated. The ink is used as soon as possible after been taken out from the ultrasonic bath.

Dropcoating

Electrodes with 50µg/cm² loading were produced. Consecutively the volume of 5 µL are pipetted twice onto the pretreated GC electrodes at room temperature and dried for 0.5 hours at 60 °C with a light uniform film formed on the electrode surface.

Electrochemical procedure

Electrochemical measurements comprise conditioning of work electrode, measurement of the open circuit potential (OCP), impedance spectroscopy (determination of iR-drop), cyclic voltammetry (CV, “activity”) and constant-potential chronamperometry (CA, “stability”). All the procedures are done in 0.1 M KOH.

Conditioning

This part of the procedure consists of the determination of the open circuit potential (OCP) for 60 s and a subsequent linear sweep from the OCP to 1 V_{RHE} (5 mV/s) to avoid a harsh potential jump and stress on the working electrode. Conditioning is done by 30 cycles at 100 mV/s from 0 V_{RHE} to 1 V_{RHE}.

Impedance spectroscopy (determination of iR-drop)

The uncompensated resistance (Ru, iR-drop) of the system is determined by electrochemical impedance spectroscopy (EIS) at the OCP. The first step is the determination of the OCP (for 60 s). EIS is carried out at the OCP: 25 data points between 100 kHz and 10 Hz with an amplitude of 10 mV_{RMS}.

Cyclic voltammetry ("activity measurement")

The cell remains switched on after EIS and the potential is swept from the OCP to 1 V_{RHE} (5 mV/s). Before start of the CV, the electrode is set to rotate at 1600 rpm. Cyclic voltammetry is conducted from 1 V_{RHE} to 1.8 V_{RHE} at 5 mV/s with automatic iR-compensation using Ru from impedance spectroscopy.

Chronoamperometry (CA)

To perform a stability test in chronoamperometric mode, the potential is kept at 1.8 V_{RHE} after stationary polarization for two hours.

ICP-OES measurement

All measurements were conducted in 1M KOH, with internal resistance (IR)-correction. A coil-shaped platinized platinum wire (PT-5W, 125 μm diameter, 99.99%, Science Products GmbH), placed along the flow channel following the electrolyte flow, was used as the counter electrode (CE), while the reference electrodes (RE) (Hg/HgO, CH Instruments Inc., CHI152, reference potential +0,098Vvs NHE) weas inserted perpendicular to the electrolyte outlet channel. All potentials are expressed vs the RHE potential scale. Catalysts were drop casted on glassy carbon support measuring a surface area of 0.196 cm^2, previously polished with fine 0.05 μm and 1.0 μm alumina powder and ultra-sonicated for 15min in MilliQ water. Catalyst inks were prepared by mixing by ultra-sonication 49% H_2O, 49% ethanol and 2% Nafion solution for 30 min. Subsequently a certain amount of catalyst ink was drop casted on the glassy carbon working electrode (WE) until a loading of 50 $\mu g/cm^2$ was achieved. The catalyst ink was dried on the glassy carbon support under an Argon stream for 30 min and finally the working electrode sample holder was inserted into the flow cell.

For all catalysts electrochemical characterization, a flow rate of 0.86ml/min was used, because it provides a good balance between oxygen gas removal from the catalyst surface and sufficient detection of the catalyst corrosion products in the ICP-OES. Lower flow rates prohibit reproducible experimental conditions due to excessive oxygen bubble formation at such high current densities. Transient signals of Co were recorded continuously with an integration interval of 100 ms and 2 sweeps per reading and detection limits are 0.12 ppb for Co according to the manufacturer. Additionally, in order to perform a background correction on the ICP-OES data a 300 s time window before and after the 2h chronopotentiometric analysis, without any current passing through the cell, was used in order to certify that no signal drift was observed. For this reason ICP-OES signal is recorded over a total time of 7800 s in contrast to the 7200 s of the chrono-potentiometric stress tests. Finally, calibration was performed using 7 standard solutions (100, 50, 10, 5, 1, 0.5 and 0 (as a blank solution) ppm metal, prepared from Merck CertiPUR®), while the RF power was set to 1400 W with a plasma gas flow rate of 15 L min^{-1}. For more information please refer here.[1]

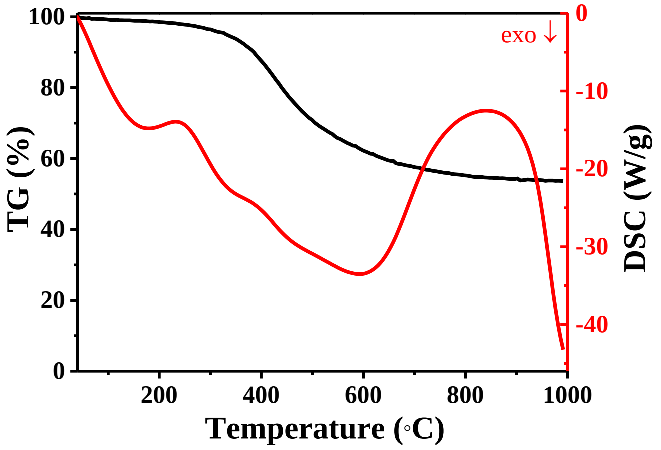

Figure S1. TG-DSC curves of the carbonization process of the carbon precursor. The data was collected under inert atmosphere with 50 mL/min argon input.

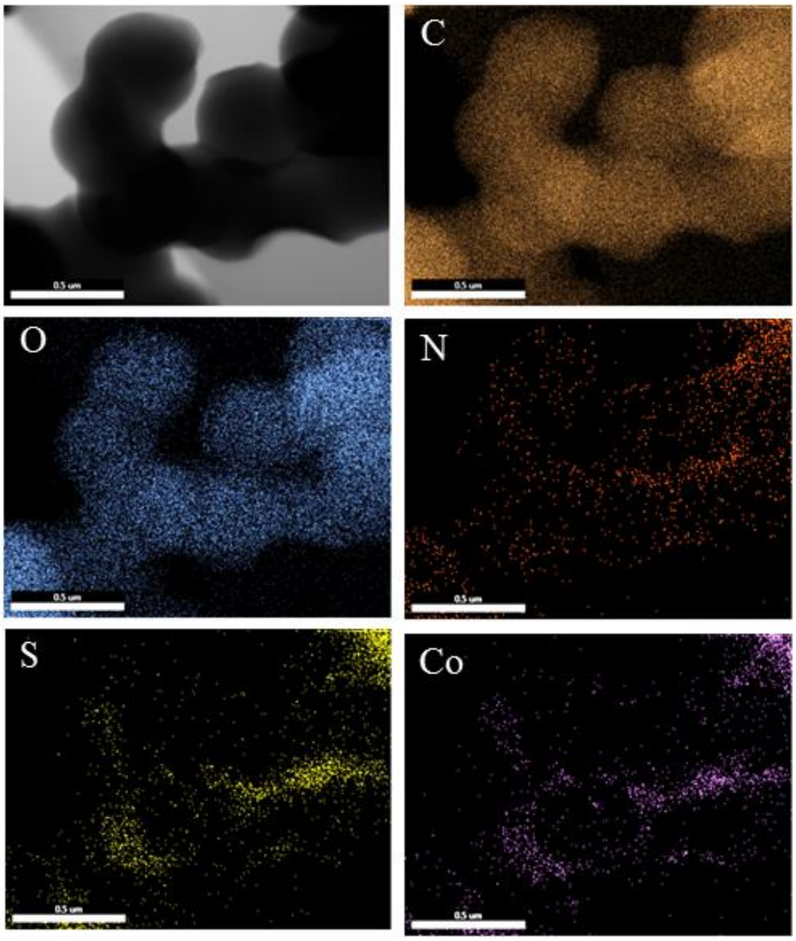

Figure S2. Mapping images of different elements (C, O, N, S and Co) from the precursor mixture of hydrothermal carbon, ionic liquid and cobalt acetate. (detected by STEM Hitachi HD-2700)

Figure S2 depicts the EDX mapping of C, O, N, S and Co within the mixtures of carbon IL and Co precursors before annealing. The carbon precursor contains only C and O elements; the N and S elements originate from the IL; the Co element comes from the Co precursor.

It can be seen that the element from IL and Co precursor are homogeneously distributed, which prove that Co is dissolved in the IL phase forming a homogeneous solution. These three elements (Co, N, S) are dispersed in the carbon precursor matrix (not everywhere). This means the combination of the IL solution and the HTC is just a physical combination and no strong interactions exist between the IL and the carbon precursor. The mixture was annealed at different temperatures under argon atmosphere to get different carbonaceous structures. It is interesting that the non-metal elements show a homogeneous distribution in annealed sample, which indicates that IL is a good candidate for the introduction of heteroatoms on the carbon surface.

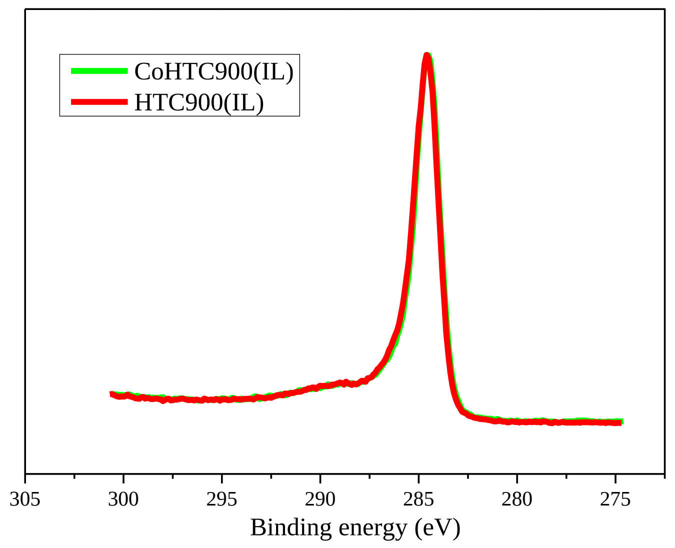

Figure S3. XPS C1s peaks of HTC900(IL) and CoHTC900(IL).

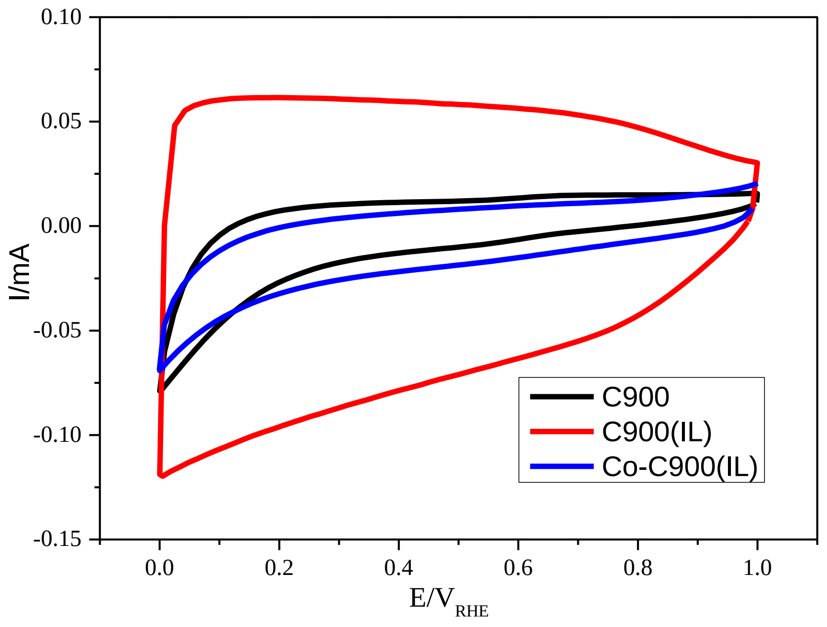

Figure S4. CV of different samples. CV is conducted from 0 V_{RHE} to 1 V_{RHE} at 100 mV/s.

Table S1. The key performance indicators (KPI) of electrochemistry data of Co-C900(IL) sample.

name	µg catalyst	geometric area	Electrolyte, PH	Protocol
CoHTC900(IL)	10	0.19625 cm^{-2}	0.1 M KOH, 13	
OCP	Ru		Initial EIS measurement	
0.875 V_{RHE}	36.2 Ω			
E_{2mA/cm^2}	E_{5mA/cm^2}	E_{10mA/cm^2}	Initial activity measurement	
1.63 V_{RHE} (auto iR-drop)	1.65 V_{RHE} (auto iR-drop)	1.68 V_{RHE} (auto iR-drop)		
$J_{m,1.6V}$	$J_{m,1.7V}$	$J_{m,1.8V}$	Mass activity in mA/µg	
0.012	0.12	0.28		
J t=0 h	J t=0.5 h	J t=2 h	Stability at 1.8 V_{RHE} (Rotating speed 1600)	
12.0 mA/cm^2	14.6 mA/cm^2	15.1 mA/cm^2		

Table S2. The key performance indicators (KPI) of electrochemistry data of Co-C900(H_2O) sample.

name	µg catalyst	geometric area	Electrolyte, PH	Protocol
CoHTC900(H_2O)	10	0.19625 cm^{-2}	0.1 M KOH, 13	
OCP	Ru		Initial EIS measurement	
0.82 V_{RHE}	42.5 Ω			
E_{2mA/cm^2}	E_{5mA/cm^2}	E_{10mA/cm^2}	Initial activity measurement	
1.64 V_{RHE} (auto iR-drop)	1.66 V_{RHE} (auto iR-drop)	1.69 V_{RHE} (auto iR-drop)		
$J_{m,1.6V}$	$J_{m,1.7V}$	$J_{m,1.8V}$	Mass activity in mA/µg	
0.006	0.09	0.24		
J t=0 h	J t=0.5 h	J t=2 h	Stability at 1.8 V_{RHE} (Rotating speed 2000)	
7.7 mA/cm^2	15.6 mA/cm^2	16.8 mA/cm^2		

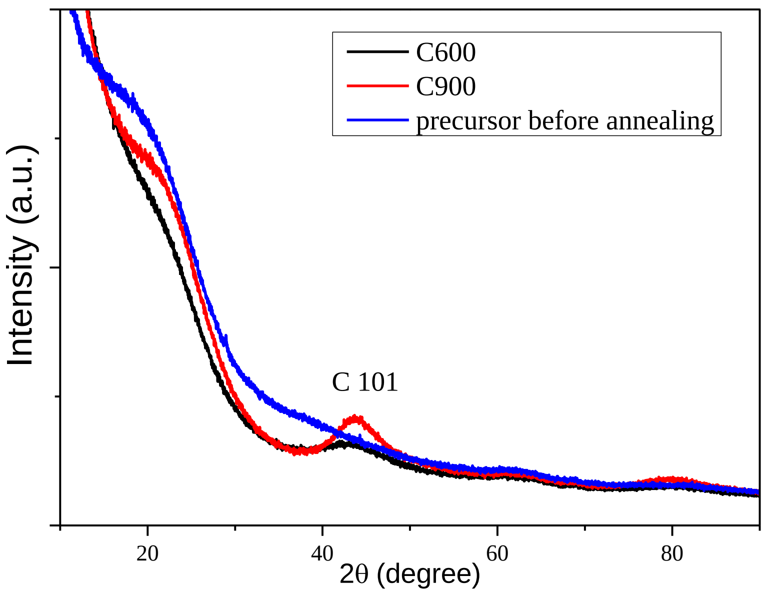

Figure S5. XRD curves of different samples.

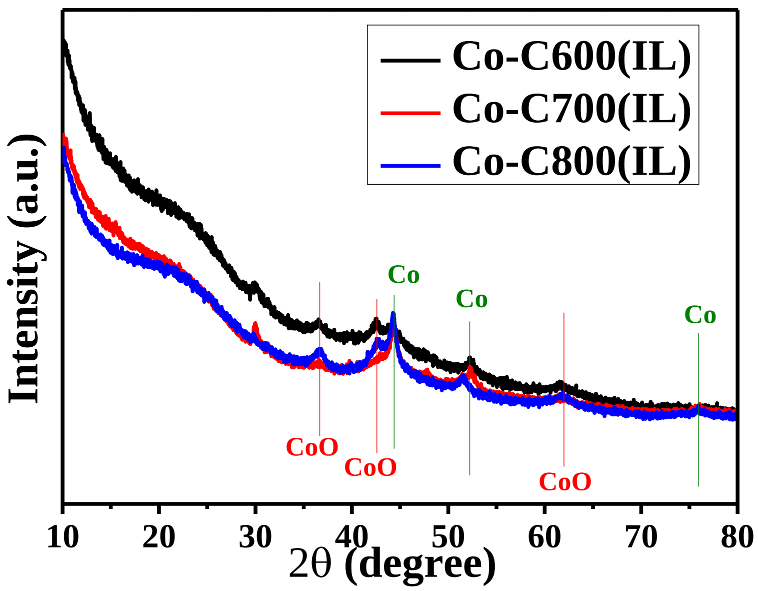

Figure S6. XRD curves of different cobalt contained samples.

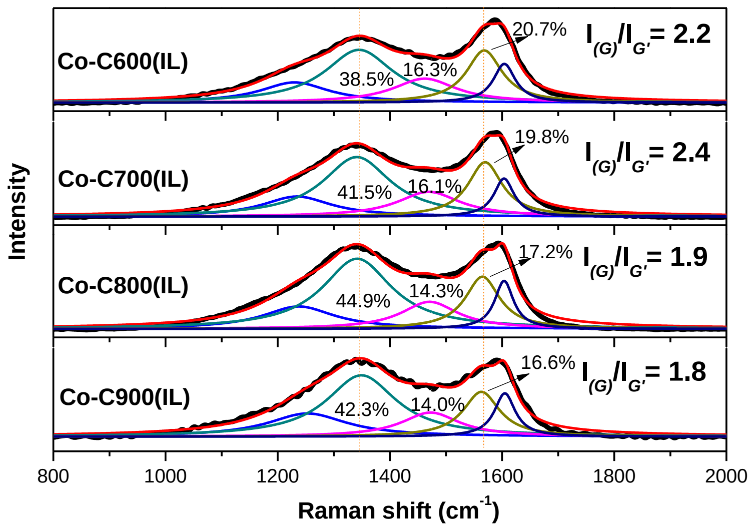

Figure S7. Raman spectra of different samples. Fitting procedure of rather amorphous carbons with 5 peaks according to Sadetzky et. al [2] was applied. The pink peak represents the contribution from amorphous carbon.

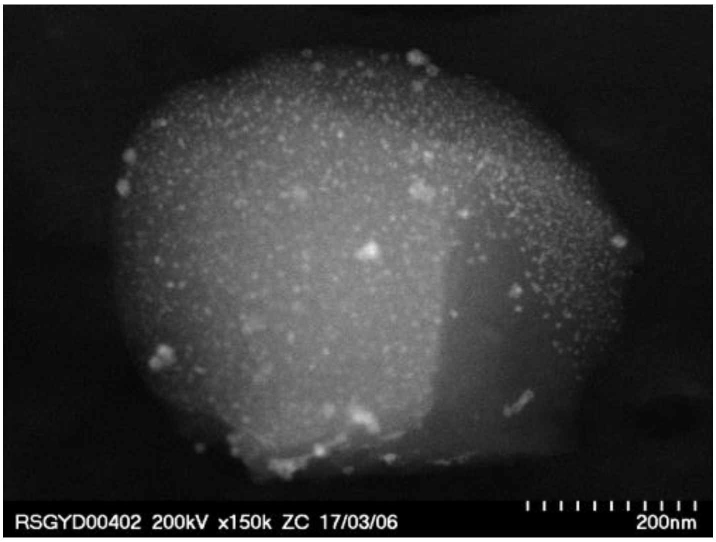

Figure S8. STEM image of the Co-C600(IL).

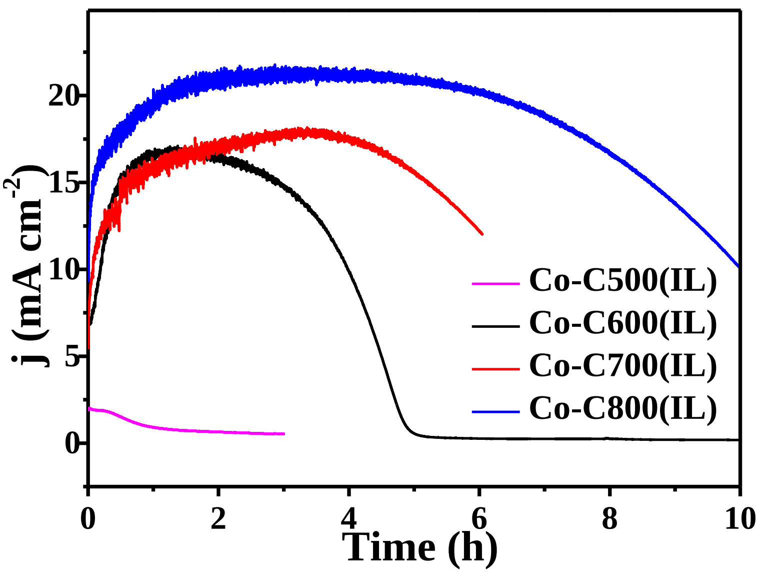

Figure S9. CA of different annealed samples. The potential is kept at 1.8 V.

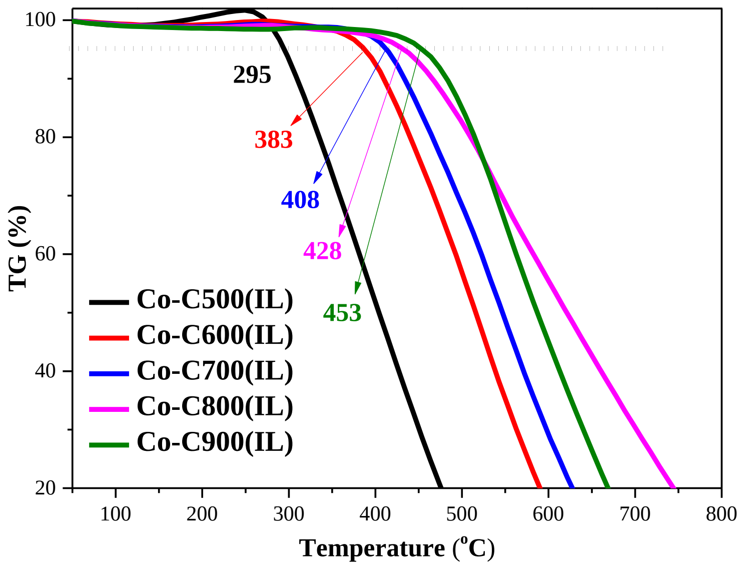

Figure S10. TG curves of different samples annealed at different temperatures in air.

Combustion process

The combustion process was conducted in TG experiments under a flow of oxygen (20% O2 in Ar balance, 50 mL min^{-1}) with a heating rate of 10 °C/min. The combustion temperature was defined at the temperature when 5% of the sample weight is lost. The sample annealed at 500 °C shows the beginning of a weight loss at relatively low temperature (295 °C, 5% loss). This is not in consequence with other samples. The reason is the 500 °C annealed sample has no carbon network formed as other samples, thus exhibiting a different combustion process.

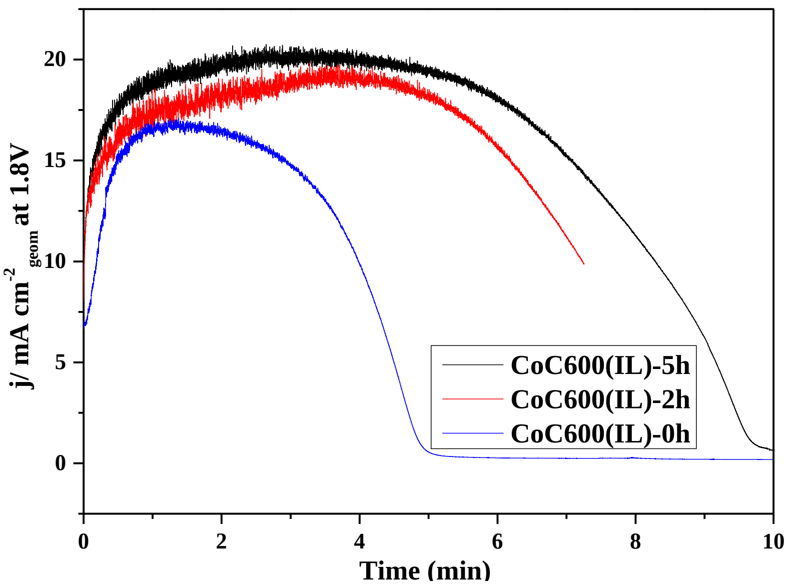

Figure S11. CA of samples annealed at 600 °C with different annealing time. The potential is kept at 1.8 V_{RHE}.

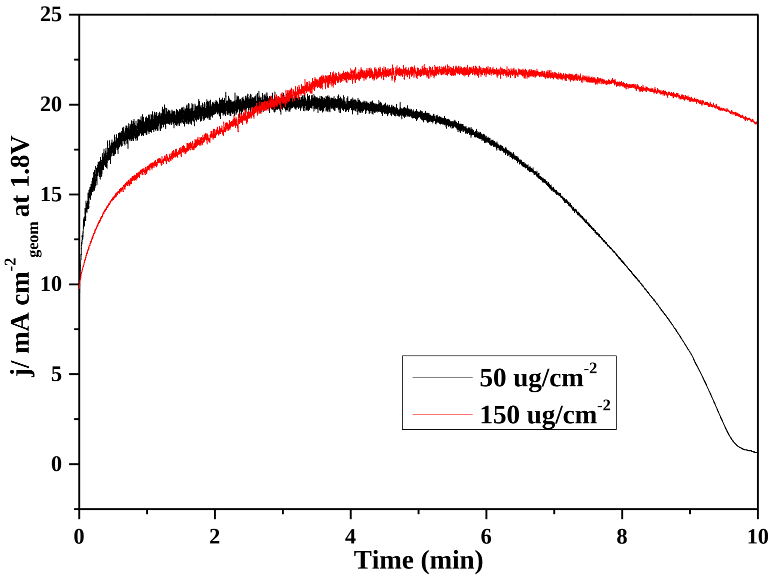

Figure S12. CA of the 600 °C annealed sample (CoSHTC-5h). The black line was determined with a sample loading content of 50 µg/cm^{-2} and the red line with a loading content of 150 µg/cm^{-2}.

References

[1] I. Spanos, A. A. Auer, S. Neugebauer, X. Deng, H. Tüysüz, R. Schlögl, *ACS Catalysis* **2017**, *7*, 3768-3778.

[2] A. Sadezky, H. Muckenhuber, H. Grothe, R. Niessner, U. Pöschl, *Carbon* **2005**, *43*, 1731-1742.
